# CDCP1 is a novel marker of the most aggressive human triple-negative breast cancers

**DOI:** 10.18632/oncotarget.11935

**Published:** 2016-09-10

**Authors:** Federica Turdo, Francesca Bianchi, Patrizia Gasparini, Marco Sandri, Marianna Sasso, Loris De Cecco, Luca Forte, Patrizia Casalini, Piera Aiello, Lucia Sfondrini, Roberto Agresti, Maria Luisa Carcangiu, Ilaria Plantamura, Gabriella Sozzi, Elda Tagliabue, Manuela Campiglio

**Affiliations:** ^1^ Molecular Targeting Unit, Fondazione IRCCS Istituto Nazionale dei Tumori, Milano, Italy; ^2^ Tumor Genomics Unit, Fondazione IRCCS Istituto Nazionale dei Tumori, Milano, Italy; ^3^ Functional Genomic Core Facility, Fondazione IRCCS Istituto Nazionale dei Tumori, Milano, Italy; ^4^ Division of Surgical Oncology, Breast Unit, Fondazione IRCCS Istituto Nazionale dei Tumori, Milano, Italy; ^5^ Division of Breast Anatomy Pathology, Fondazione IRCCS Istituto Nazionale dei Tumori, Milano, Italy; ^6^ Start-Up Unit, Fondazione IRCCS Istituto Nazionale dei Tumori, Milan, Italy; ^7^ Dipartimento di Scienze Biomediche per la Salute, Università degli Studi di Milano, Milan, Italy

**Keywords:** triple-negative breast cancer, CDCP1, CDCP1 copy number, prognosis, metastasis

## Abstract

CDCP1, a transmembrane noncatalytic receptor, the expression of which has been associated with a poor prognosis in certain epithelial cancers, was found to be expressed in highly aggressive triple-negative breast cancer (TNBC) cell models, in which it promoted aggressive activities—ie, migration, invasion, anchorage-independent tumor growth, and the formation of vascular-like structures *in vitro*. By immunohistochemical (IHC) analysis of 100 human TNBC specimens, CDCP1 was overexpressed in 57% of samples, 38% of which exhibited a gain in CDCP1 copy number by fluorescence *in situ* hybridization (FISH). CDCP1 positivity was significantly associated between FISH and IHC. CDCP1 expression and gains in *CDCP1* copy number synergized with nodal (N) status in determining disease-free and distant disease-free survival. The hazard ratios (HRs) of the synergies between CDCP1 positivity by IHC and FISH and lymph node positivity in predicting relapse did not differ significantly, indicating that CDCP1 overexpression in human primary TNBCs, regardless of being driven by gains in *CDCP1*, is for a critical factor in the progression of N-positive TNBCs. Thus, CDCP1 is a novel marker of the most aggressive N-positive TNBCs and a potential therapeutic target.

## INTRODUCTION

Triple-negative breast cancers (TNBCs) are highly aggressive, with early recurrence and leading to high mortality rates within the first several years postdiagnosis [1, 2]. Molecular profiling of TNBC specimens in the last decade has demonstrated the heterogeneity of this disease and has led to the identification of many TNBC subtypes [3–6], implicating several molecules as drivers of each subclass and thus as targets for therapy [4, 6]. However, none of these molecular classifications has significantly improved the clinical management of TNBC, with the exception of androgen receptor-positive [7] and BRCA-1-deficient TNBC, for which existing therapies are promising [8]. Thus, the identification of specific markers of the aggressiveness of TNBCs that can be targeted for therapy remains a challenge.

CDCP1 is a transmembrane, noncatalytic receptor that was discovered by Scherl-Mostageer [9] and later isolated using an approach that was designed to identify proteins that were involved in metastasis [10]. Its expression is associated with the loss of anchorage in epithelial cells during mitosis under physiological and pathological conditions [11], rendering tumor cells resistant to anoikis [12]. High membrane CDCP1 levels have been associated with a poor prognosis in epithelial tumors, such as lung, pancreatic, colorectal, renal, and ovarian carcinomas [13–17], but are linked to favorable prognoses in endometrial [18] and esophageal [19] carcinomas. CDCP1 mRNA and protein have been reported to be overexpressed in large cohorts of primary breast tumors and increase further in metastases [20]. Recently, *CDCP1* mRNA levels were found to rise in TNBCs and the mechanism by which CDCP1 effects aggressiveness has been suggested in cellular models [21, 22].

In this study, by stimulating TNBC cells with postsurgery wound-healing fluids (WHFs) from breast cancer patients to mimic a protumorigenic microenvironment, we identified CDCP1 as the only membrane receptor that was significantly upregulated. CDCP1 expression confers features of aggressiveness in TNBC cell lines and is significantly associated with shorter disease-free survival (DFS) and distant DFS (DDFS) in human primary TNBCs.

## RESULTS

### A protumor igenic microenvironment significantly upregulates CDCP1 mRNA and protein in TNBC cell models

The postsurgery wound-healing process can have protumor activity due to the enrichment in small molecules [23] that are needed for healing and can influence tumor cell survival and progression ([24] and references therein). Thus, we used postsurgery wound-healing fluids (WHFs) from breast cancer patients (Supplementary Table S1) to identify membrane surface molecules that were upregulated by the healing protumorigenic microenvironment and possibly involved in the progression of TNBC, rendering them therapeutic targets.

**Figure 1:**
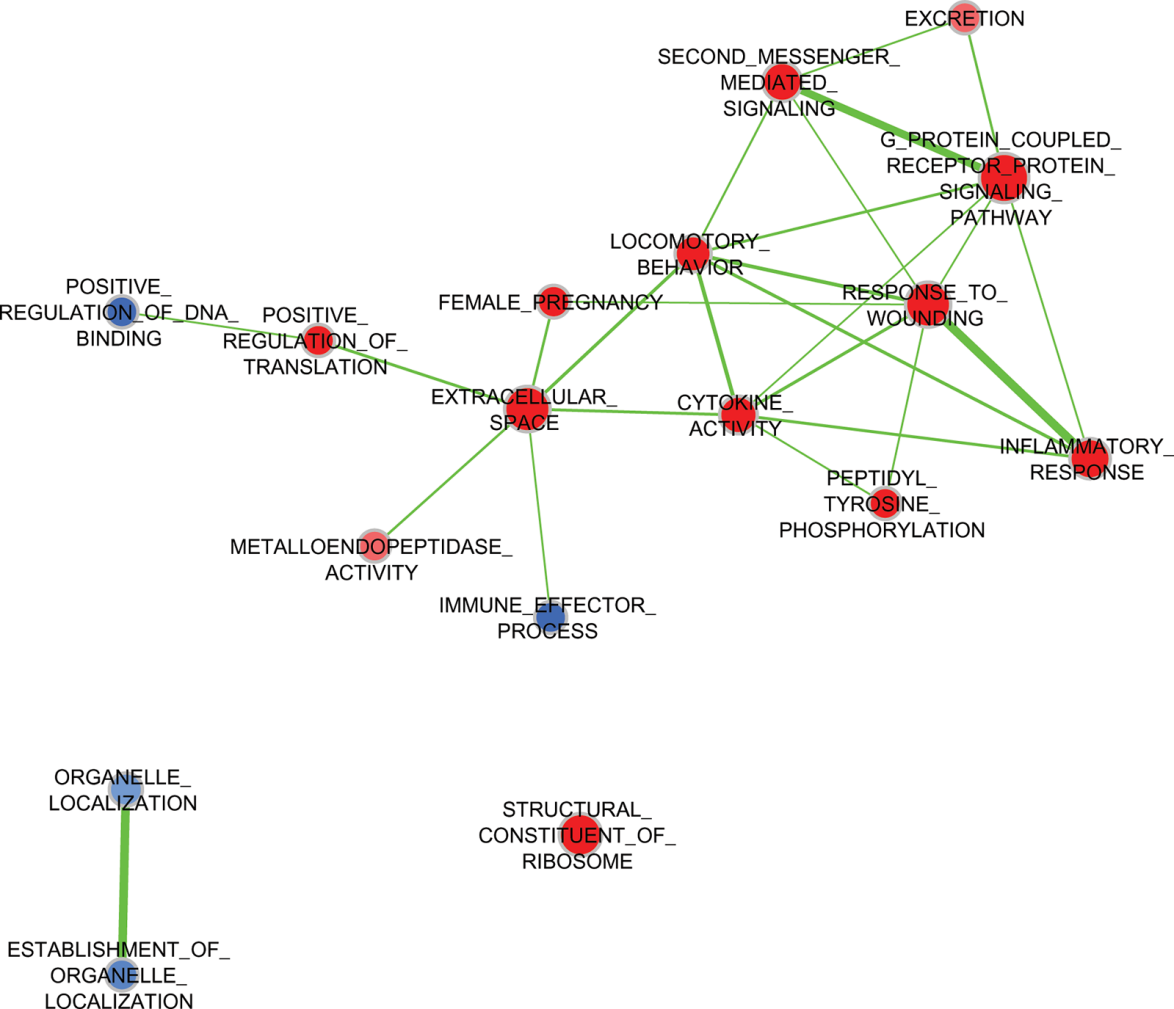
Expression of gene pathways by treatment with WHFs Enrichment Map of pathways (Gene Ontology Biological Processes) significantly enriched (negative enrichment, blue; positive enrichment, red) (FDR < 0.2) in WHF-treated compared to untreated TNBC cell lines by GSEA analysis.

Five TNBC cell lines—MDA-MB-231, BT-549, and MDA-MB-157 (classified molecularly as mesenchymal-like) and MDA-MB-468 and SUM149 (considered to be basal-like [4])—were starved, treated with pooled WHFs for 48 h or left untreated, and profiled using the Illumina platform. The experimental design comprised a training set and subsequent validation of an independent set of cells that were treated with WHFs or not (see Materials and Methods). Enrichment analysis according to WHF treatment identified several gene sets differentially and significantly enriched in the WHF-treated cells characterized by over expression of genes related to response to wound, second messenger- and G protein-mediated-signaling, cytokine activity and locomotory characteristics (Figure 1), supporting the appropriateness of our approach in identifying mediators of TNBC aggressiveness. To identify plasma membrane receptors that were upregulated in TNBC models that correlated with TNBC aggressiveness and were potential therapeutic targets, we focused on molecules that resided on the cell membrane surface (see Materials and Methods). Based on a false discovery rate (FDR) < 0.1 and a fold-change >2, by class comparison analysis of all cell lines, *CDCP1* was the only gene that increased significantly after WHF stimulation, with a geometric mean fold-change in mRNA of 4.17 (0.0068) and 3.38 (*p* = 0.0077) in the training and validation sets, respectively. To confirm this rise in *CDCP1* mRNA, *CDCP1* transcripts were amplified by TaqMan assay. As shown in Table 1, CDCP1 mRNA was significantly upregulated after WHF treatment with both probes.

**Table 1:** CDCP1 mRNA and protein increase upon 48 h of WHF treatment

TNBC cell line	Gene expression by microarray Log fold-change in CDCP1 gene expression^a^		Gene expression by RT-qPCR				Fold-change in CDCP1 protein expression^c^ (mean ± SEM^d^)
			ΔΔ Ct (*CDCP1 in* WHF-treated and untreated cells)^b^				
	T^e^	V^e^	Hs01080405_m1		Hs01080410_m1		
			T	V	T	V	
MDA-MB-231	4.13	3.79	1.32	2.61	1.32	2.66	1.29 (± 0.09)
BT549	3.20	1.9	2.81	2.11	4.18	2.86	1.25 (± 0.08)
SUM 149	2.10	2.64	2.37	1.08	1.4	−0.26	3.60 (± 1.42)
SUM 159	na^f^	na	na	na	na	na	2.65 (± 1.19)
HCC1937	na	na	na	na	na	na	1.61 (± 0.53)
MDA-MB-157	0.62	0.395	na	na	na	na	nd^g^
MDA-MB-468	0.12	0.066	2.2	2.14	2.22	1.94	0.81 (± 0.24)
Geometric Mean (*p* value)	4.17 (0.0068)	3.38 (0.0077)	4.51 (0.0061)	3.96 (0.0086)	4.86 (0.042)	3.34 (0.086)	
Average protein fold change (*p* value)							1.87 (0.0696^h^)

a Fold-change was calculated in the training and validating sets between the normalized gene expression levels of *CDCP1* in TNBC cell lines treated with WHFs for 48 h *versus* untreated.

b *CDCP1* mRNA levels were analyzed using the “best covering” probe Hs01080405_m1 and the probe Hs01080410_m1, as indicated by Thermo Fisher Scientific.

c Fold-change in CDCP1 protein was calculated based on relative quantification of western blot signals of full-length CDCP1 normalized to corresponding actin signals in TNBC cell lines treated with WHFs for 48 h *versus* untreated.

d Mean ± standard error of the mean in at least 3 independent experiments.

e T, training set; V, validation set.

f na, not available.

g nd, not detectable.

h Student's *t*-test of normalized fold-change of CDCP1 protein expression in each cell lines.

**Figure 2:**
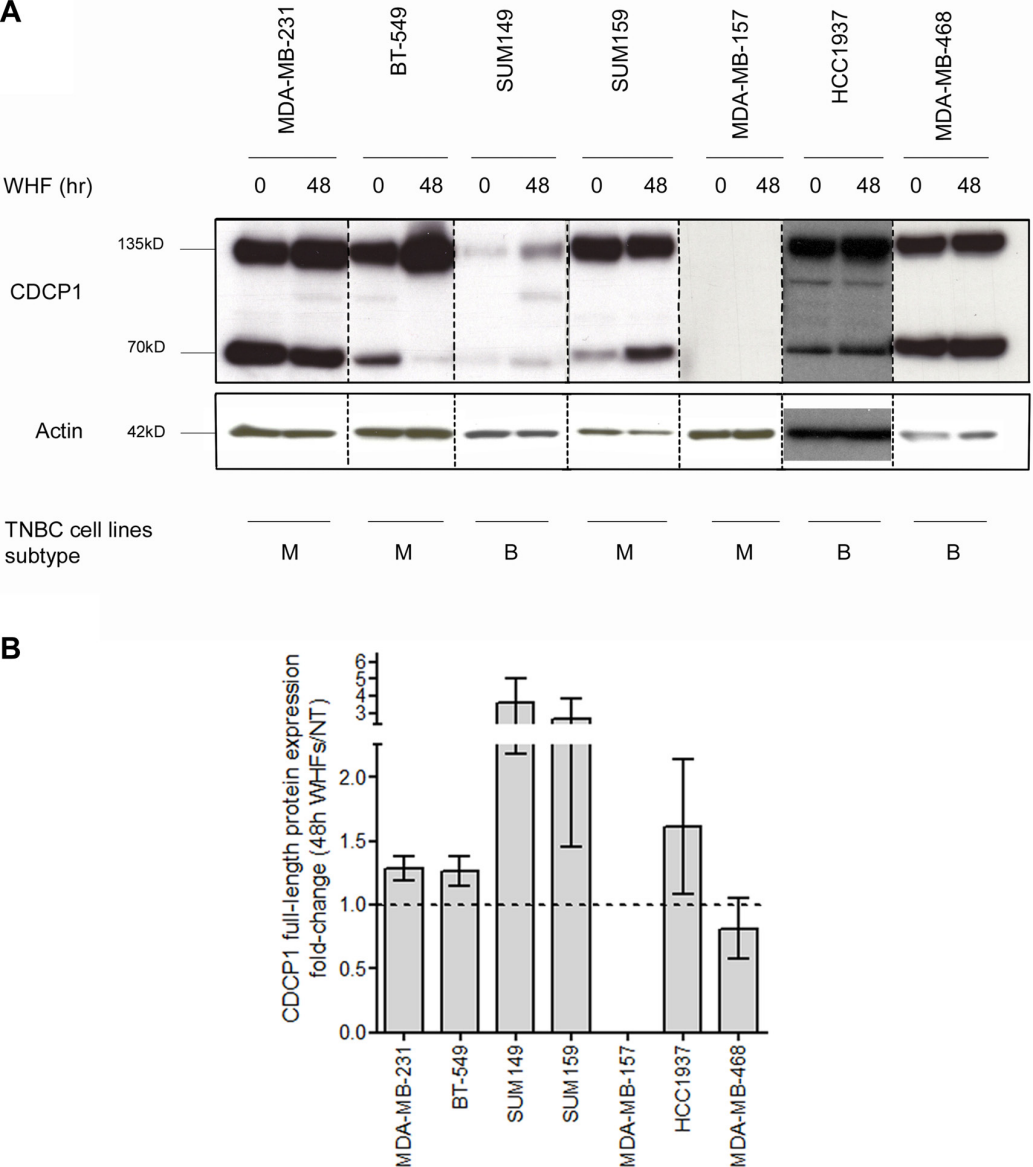
Regulation of CDCP1 expression by WHF in TNBC cell lines (**A**) The TNBC cell lines MDA-MB-231, BT-549, SUM149, SUM159, MDA-MB-157, HCC1937 and MDA-MB-468, considered basal-like (B) or mesenchymal-like (M) per Lehmann classification (4), were starved (0% FBS) for 24 h, stimulated for 48 h with a WHF pool, and processed for western blot analysis of CDCP1 (the full-length 135-kD and 70-kD forms) using polyclonal anti-CDCP1. Monoclonal anti-actin was used as a loading control. The results are representative of 3 independent experiments. (**B**) The graph shows the fold-increase ± standard error of the mean (SEM) in full-length CDCP1 on WHF stimulation for each cell line in 3 western blot experiments, evaluated by densitometry and normalized to actin levels.

We then performed western blot to determine whether the elevation in *CDCP1* mRNA was mirrored by greater CDCP1 expression, detecting the 135-kD full-length and 70-kD cleaved membrane-bound forms—which coexist in human tumors [25]—in 6 of the 7 TNBC cell lines, independent of WHF treatment (Figure 2A). On WHF stimulation using the same treatment schedule as in the gene expression profiling experiment, full-length CDCP1 rose in all cell lines except MDA-MB-468 and MDA-MB-157, the latter of which did not express CDCP1 at baseline or under WHF stimulation conditions (Figure 2A, 2B and Table 1). No differences were noted in the induction of CDCP1 between the mesenchymal-like and basal-like subtypes (Figure 2A, 2B). The 70-kD form of CDCP1 increased only on WHF treatment in the SUM149 and SUM159 cell lines (Supplementary Figure S1).

### CDCP1 promotes features of aggressiveness in TNBC cell lines

**Figure 3:**
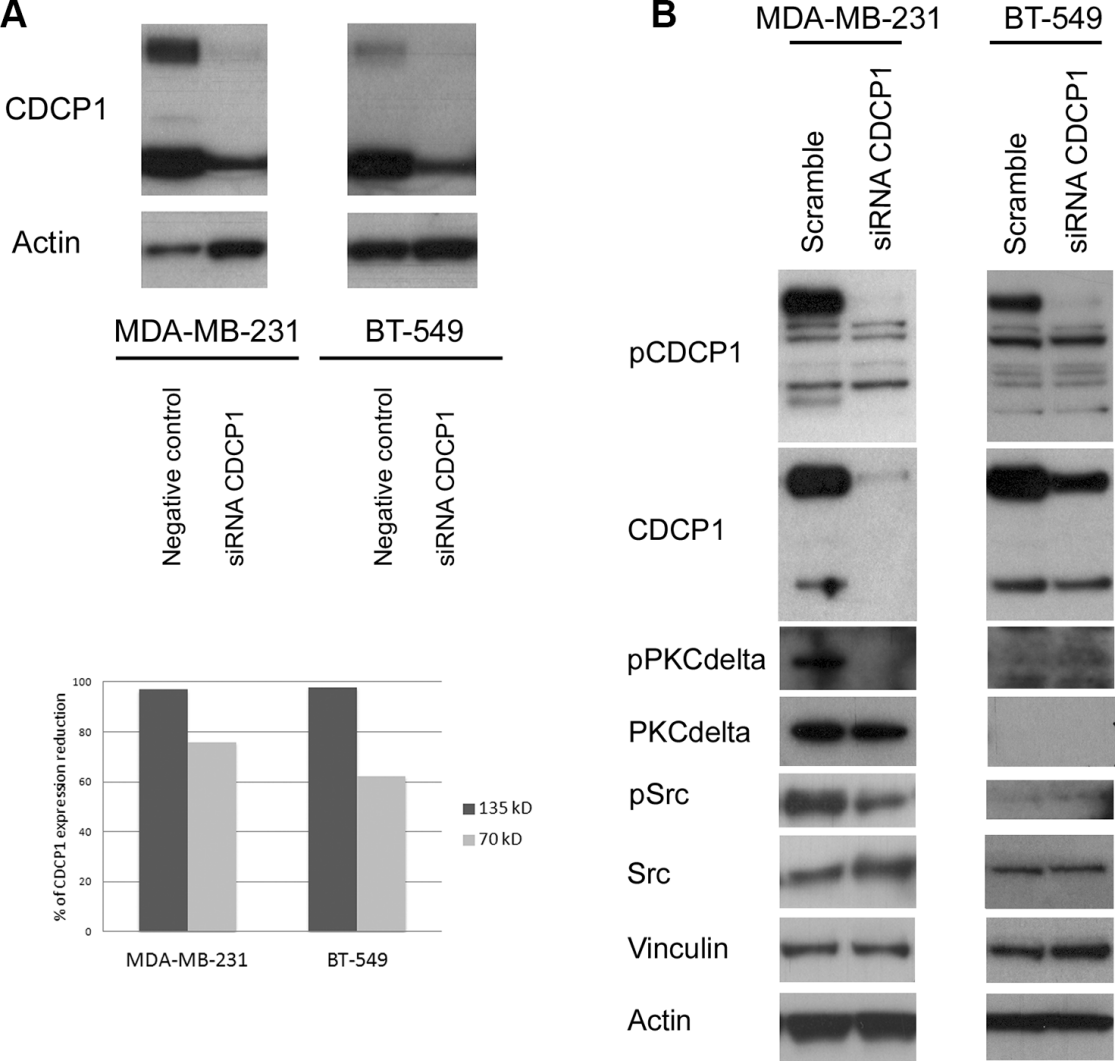
Effect of silencing CDCP1 on signaling mediators (**A**) MDA-MB-231 and BT-549 cells were transfected with a pool of 4 oligonucleotides (100 nM) that bind and degrade *CDCP1* mRNA (see Materials and Methods for sequences) or with the appropriate negative control siRNAs. Cells were harvested at 48 h post-transfection, and CDCP1 expression was verified by western blot. Monoclonal anti-actin was used as a loading control. The knockdown efficiency was ≥ 60% for both CDCP1 forms. (**B**) CDCP1 siRNA-treated and control-treated MDA-MB-231 and BT-549 cells were analyzed for activation of CDCP1, Src, and PKCδ. Monoclonal anti-actin and anti-vinculin were used as loading controls.

**Figure 4:**
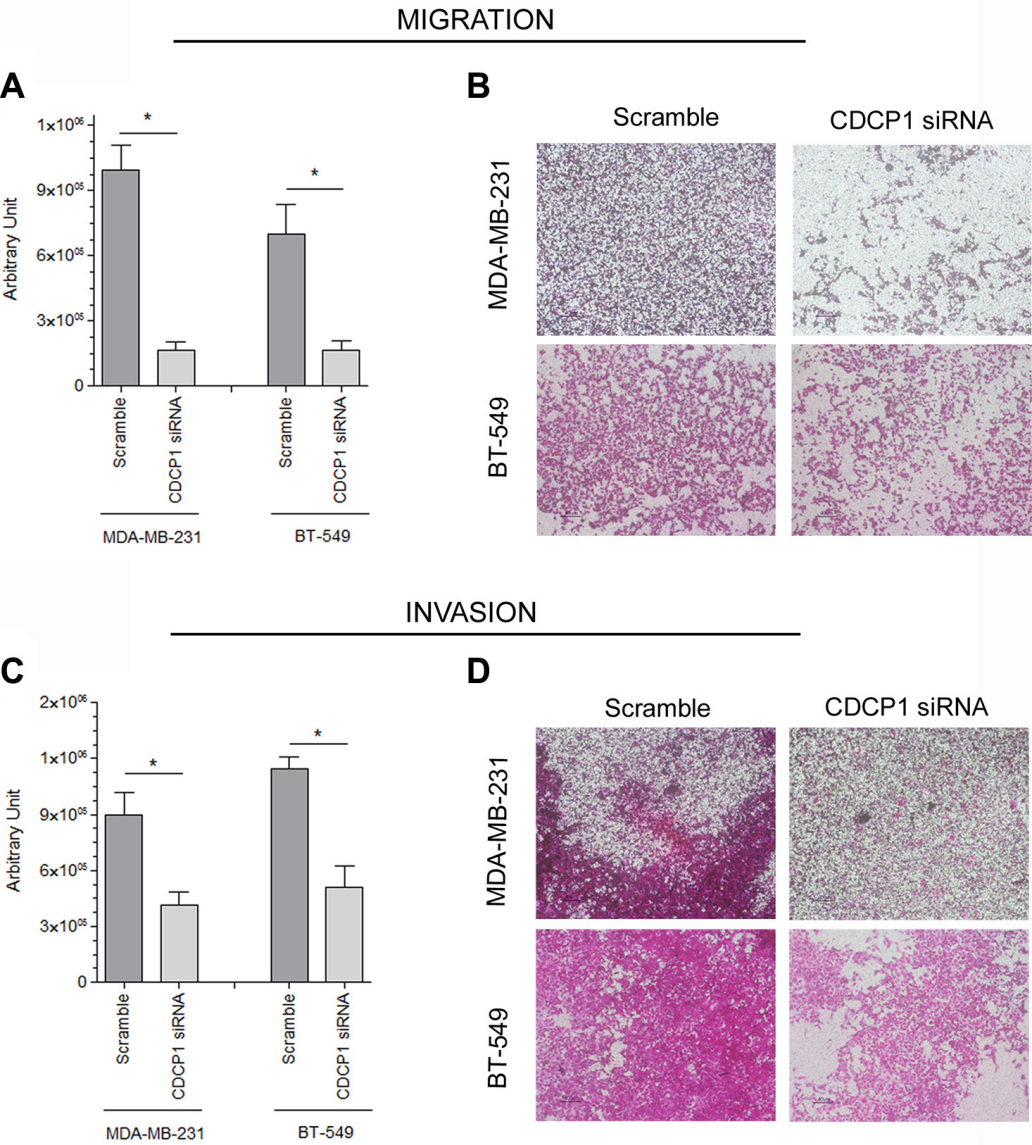
Correlation between CDCP1 and migration and invasion in TNBC cell lines CDCP1 siRNA-treated and control-treated MDA-MB-231 and BT-549 cells were plated into Boyden chambers for the migration assay (**A**, **B**) and in Transwells that were coated with Matrigel for the invasion assay (**C**, **D**). The area occupied by migrated cells in the Transwell assay (mean ± SEM in 3 independent experiments) was measured by digital image analysis (Image Pro-Plus 7.0 application, Media Cybernetics). *p < 0.05.

**Figure 5:**
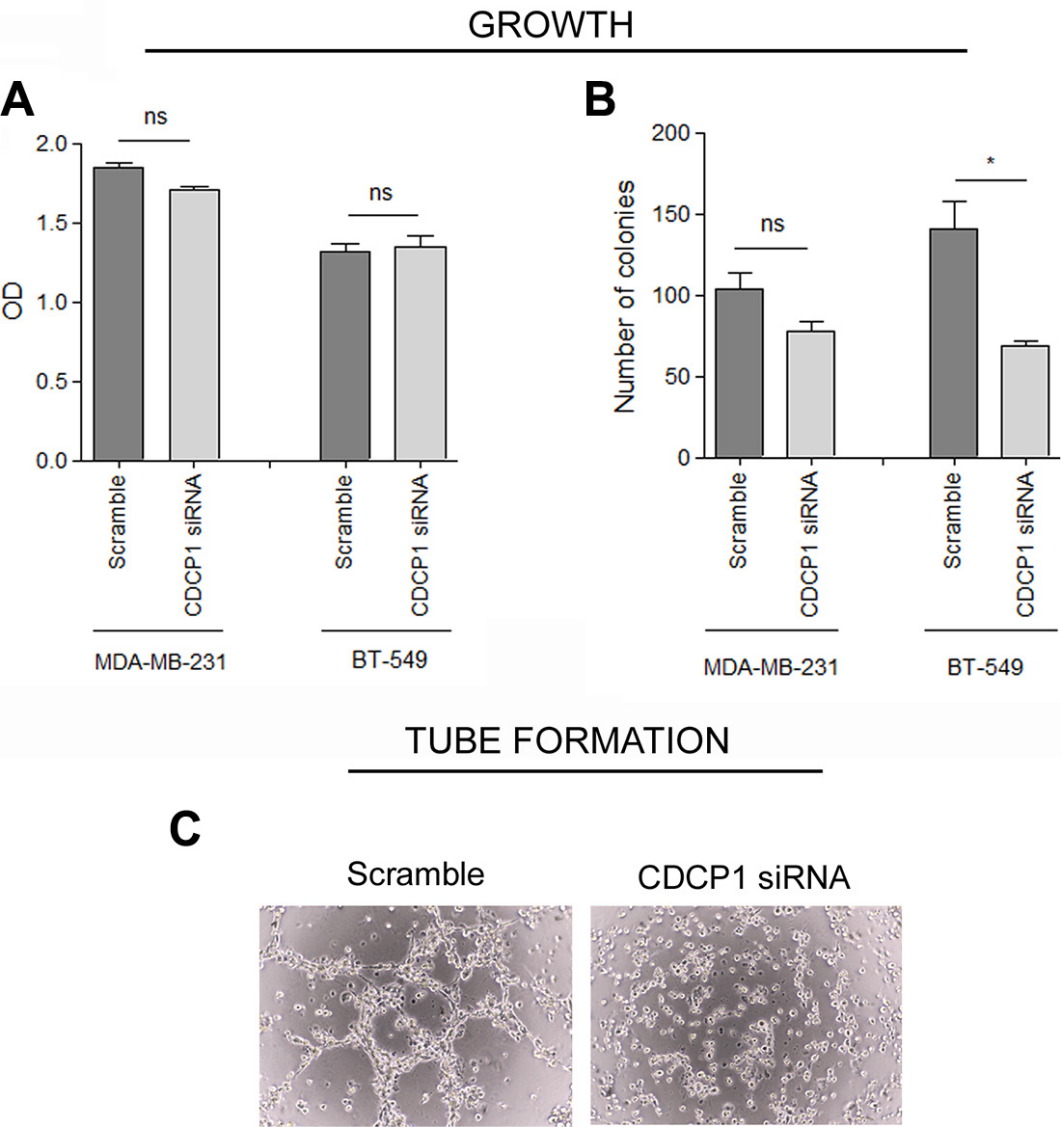
CDCP1 correlation with growth of TNBC cell lines (**A**) Proliferation under anchorage-dependent conditions (2D) of CDCP1 siRNA- and control siRNA-treated cells was evaluated by SRB (mean ± SEM in 3 independent experiments). (**B**) Anchorage-independent tumor growth (3D) was performed as described in Materials and Methods on MDA-MB-231 and BT-549 cells expressing CDCP1 or in which CDCP1 was knocked down. Colony growth was determined by counting all colonies in each well 15 days after seeding at 40x magnification. (**C**) Tube formation assay was performed as described in Materials and Methods on MDA-MB-231 cells expressing CDCP1 or in which CDCP1 was knocked down. *p < 0.05; ns, not significant.

To determine whether CDCP1 has prometastatic activity, as suggested for certain human cancer histotypes [13–17], we analyzed the *in vitro* migration, invasion, and proliferation of 2 TNBC cell lines (BT-549 and MDA-MB-231) that highly express CDCP1 (Figure 2A), migrate, and invade, after silencing CDCP1. In both lines, the efficiency of CDCP1 knockdown exceeded 60% for both forms (Figure 3A), and consequently, CDCP1 activation was reduced. Moreover, Src and PKCδ, mediators of CDCP1 signaling [26], underwent less phosphorylation only in MDA-MB-231 cells (Figure 3B). Knockdown of CDCP1 inhibited migration by 84% in MDA-MB-231 cells (*p* = 0.0196) and by 76% in the BT-549 line (*p* = 0.0465) (Figure 4A, 4B). Similarly, depletion of CDCP1 impeded the invasion of both cell lines (Figure 4C, 4D), declining by 54% in MDA-MB-231 cells (*p* = 0.0173) and 56% in BT-549 (*p* = 0.0179) cells. CDCP1 knockdown did not alter the growth of MDA-MB-231 and BT-549 cells considerably in 2D cultures (Figure 5A), while the decrease in growth in 3D cultures was significant in BT-549 cells (Figure 5B).

We then examined whether CDCP1 affects the ability of TNBC cells to form vascular-like structures *in vitro*—another feature of aggressiveness that we recently reported in TNBC cell lines and in human TNBC specimens [27]—compared with other breast cancer subtypes. Silencing of CDCP1 in MDA-MB-231 cells abrogated their ability to form completely closed loops (Figure 5C), suggesting that CDCP1 mediates the acquisition of vasculogenic-like networks.

### Human TNBCs express CDCP1

We analyzed the expression of CDCP1 in a cohort of 100 human primary TNBC specimens. Of these patients (median age, 53 years; range 26–84 years), 42.0% was node (N)-positive and 58.6% of tumors were stage T1. As expected for TNBCs, the tumors were primarily grade III (85.7%) and highly necrotic (80.4%). Multifocality was present in 22.6% of cases, and 36.8% of patients had ductal carcinoma *in situ* (DCIS).

**Figure 6:**
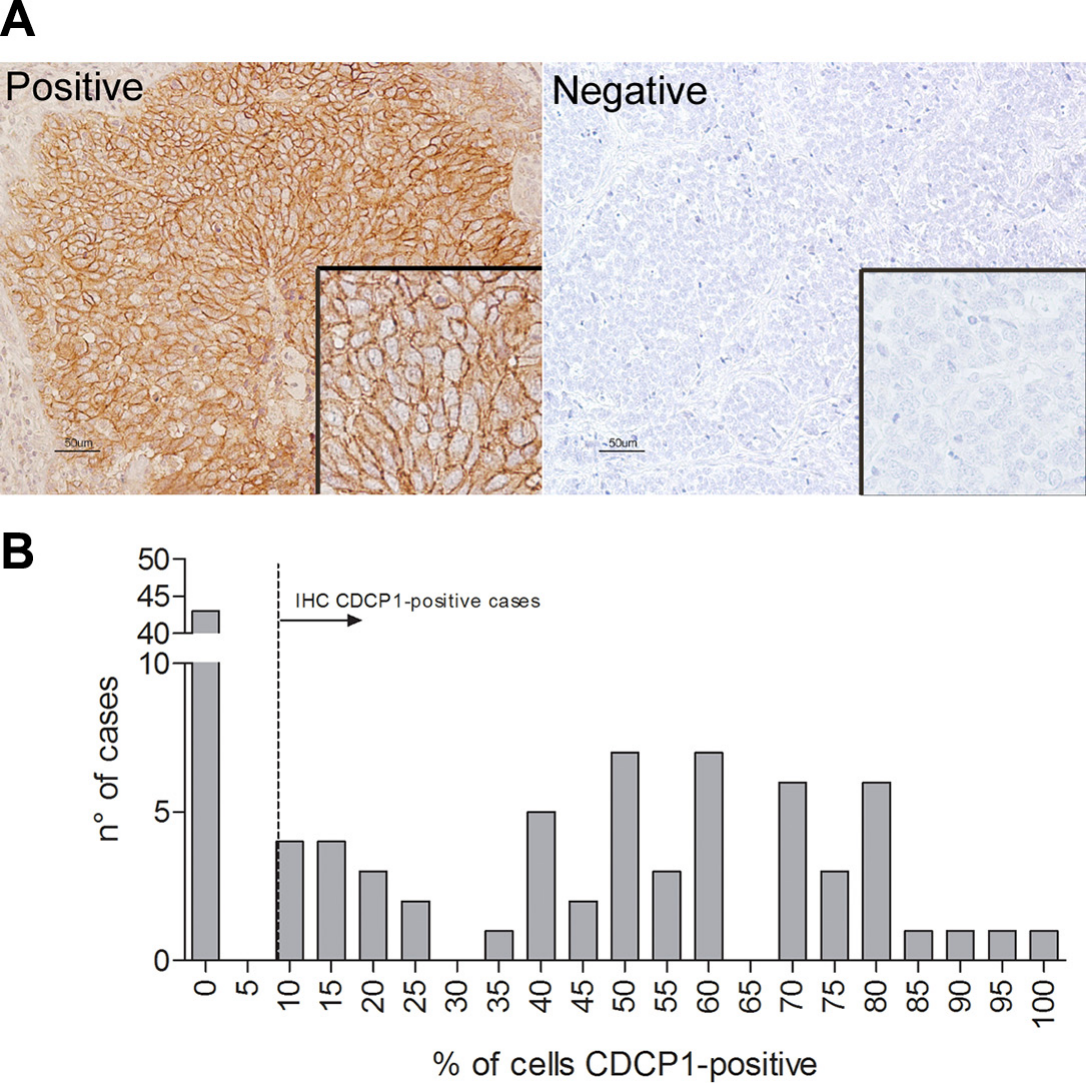
CDCP1 expression in human primary TNBCs (**A**) FFPE TNBC sections were immunostained with polyclonal anti-CDCP1 (Thermo Fisher Scientific), and membrane staining intensity was scored as positive or negative as described in Materials and Methods. Scale bar: 50 μm (insets 2×). (**B**) Frequency distribution of human specimens by percentage of CDCP1-positive cells.

Formalin-fixed, paraffin-embedded (FFPE) human TNBC sections were analyzed by immunohistochemistry (IHC) with an antibody against CDCP1. Tumors were considered positive when ≥ 10% of tumor cells showed membrane reactivity (Figure 6A) (mean CDCP1-positive tumor cells/section = 52%, range 10% to 100%); 57% (57/100) of cases were CDCP1-positive, 63% (36/57) of which expressed CDCP1 in ≥ 50% of tumor cells (Figure 6B).

**Figure 7:**
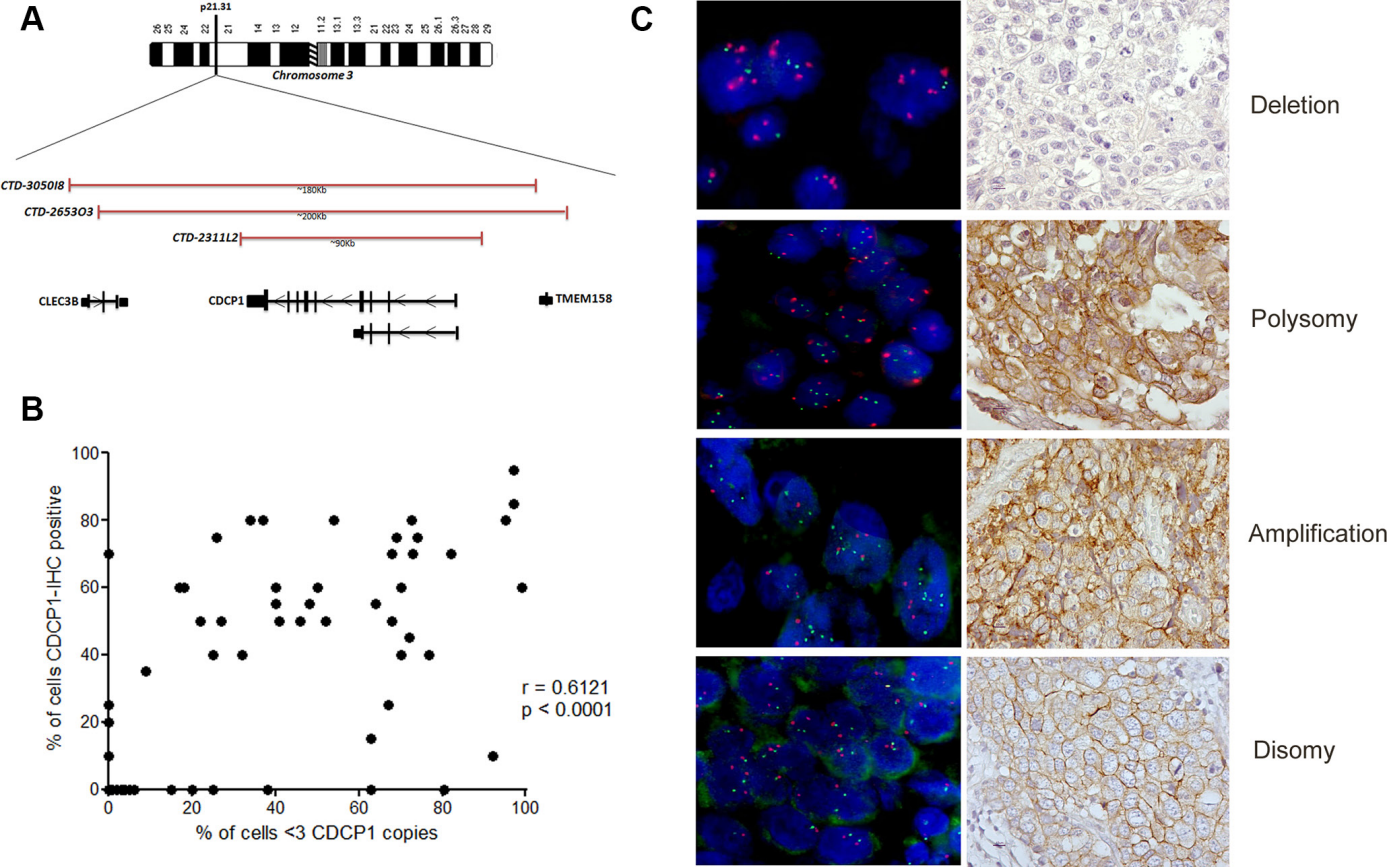
Genetic alterations in CDCP1 in human primary TNBCs (**A**) Physical map of 3p21.31 region (spanning *CDCP1*), including genomic clones selected for FISH experiments covering the *CDCP1* locus, their size in Kb, and the genes that they encompass. Maps are derived from University of California Santa Cruz Genome Browser (http://genome.ucsc.edu/), with adaptations. (**B**) Representative images of dual-color FISH using *CDCP1*/CEP3 probes on FFPE human primary TNBC specimens, showing tumor cells with: > 3 red signals for CEP3 and > 3 green signals for the *CDCP1* locus (polysomy); < 3 signals for CEP3 and > 3 green signals for *CDCP1* (amplification); < 3 signals for CEP3 and < 3 green signals for *CDCP1* (disomy); and > 3 signals for CEP3 and < 3 green signals for the *CDCP1* locus (deletion). (**C**) Correlation analysis between percentage of FISH-positive cells with percentage of IHC-positive cells (*n* = 75; *p* < 0.0001; Pearson r = 0.6121; 95% confidence interval 0.4472 to 0.7367).

*CDCP1* status in 75 of 100 available TNBC specimens was analyzed by fluorescence *in situ* hybridization (FISH) using a pool of 3 bacterial artificial chromosome (BAC) clones (BAC 2311L2, BAC 265303, and BAC 3050I8) that encompassed *CDCP1* at chromosome 3p21.31 (Figure 7A and Supplementary Figure S2). By FISH analysis, we identified 4 genetic categories: 1) disomic, with 2 copies of *CDCP1* and a centromere (*CDCP1* < 3, CEP3 < 3) (50/75, 67%); 2) amplified (*CDCP1* ≥ 3, CEP3 < 3 in at least 10% of tumor cells) (4/75, 5%); 3) polysomic (*CDCP1* ≥ 3, CEP3 ≥ 3 in at least 40% of tumor cells) (15/75, 20%); and 4) *CDCP1* deleted with respect to its centromere (*CDCP1* < 3 CEP3 ≥ 3) (6/75, 8%). Figure 7B shows representative FISH and IHC images of CDCP1 in the same cases. Polysomy of *CDCP1* was not a random event that was driven by generalized polysomy, as demonstrated by FISH analysis of all *CDCP1*-positive cases using a centromeric probe for chromosome 2 (data not shown). *CDCP1* amplification and polysomy were considered FISH-positive for gene acquisition, while the other two categories were considered FISH-negative for gene gain.

The FISH and IHC results were significantly associated—among the 19 positive cases by FISH- 17 (89.5%) were CDCP1-positive by IHC, whereas 28 (50.0%) of the 56 FISH-negative cases were CDCP1-positive by IHC (*p* = 0.003). Thus, a gain in *CDCP1* appeared to drive CDCP1 overexpression in 38% of CDCP1 IHC-positive TNBCs (17/45 cases). The percentage of cells that were positive for CDCP1 by IHC correlated with the percentage of cells that harbored more than 3 *CDCP1* signals per cell (*r* = 0.6121; *p* < 0.0001; Figure 7C). CDCP1-positive cases by IHC or FISH did not differ significantly with regard to the variables in Table 2 compared with their CDCP1-negative counterparts.

### Overexpression of CDCP1 defines a subset of human TNBC cases with a poorer prognosis

**Table 2:** Clinicopathological characteristics by CDCP1-IHC and CDCP1-FISH positivity

Characteristic	IHC		*p*^a^	FISH		*p*^a^
	CDCP1-neg No./total (%)	CDCP1-pos No./total (%)		CDCP1-neg No./total (%)	CDCP1-pos No./total (%)	
Age ≥ 50 yr	21/30 (70.0)	26/45 (57.8)	0.3356	33/56 (58.9)	14/19 (73.7)	0.7891
N-positive	9/30 (30.0)	22/45 (48.9)	0.1510	20/56 (35.7)	11/19 (57.9)	0.1104
Tumor size > 2.0 cm	9/30 (30.0)	21/45 (46.7)	0.2288	20/56 (35.7)	10/19 (52.6)	0.2785
Grade III	26/30 (86.7)	39/45 (86.7)	1.0000	48/56 (85.7)	17/19 (89.5)	1.0000
Multifocality	5/30 (16.7)	14/41 (34.2)	0.1138	12/54 (22.2)	7/17 (41.2)	0.2070
DCIS^b^	11/30 (36.7)	19/43 (44.2)	0.6305	20/55 (36.4)	10/18 (55.6)	0.1759
Necrosis	23/30 (76.7)	37/43 (86.0)	0.3595	43/55 (78.2)	17/18 (94.5)	0.1647
Ki-67-positive^c^	20/26 (76.9)	38/42 (90.5)	0.1646	41/49 (83.7)	17/19 (89.5)	0.7137
CK5/6-positive^d^	21/29 (72.4)	30/43 (69.8)	1.0000	38/55 (69.1)	13/17 (76.5)	0.7618

a Evaluated by Fisher exact test.

b DCIS, ductal carcinoma *in situ.*

c Ki-67-positive, Ki-67 > 14%.

d CK5/6-positive, cytokeratin 5/6-positive if any cytoplasmic and/or membranous invasive carcinoma cell staining was observed.

**Figure 8:**
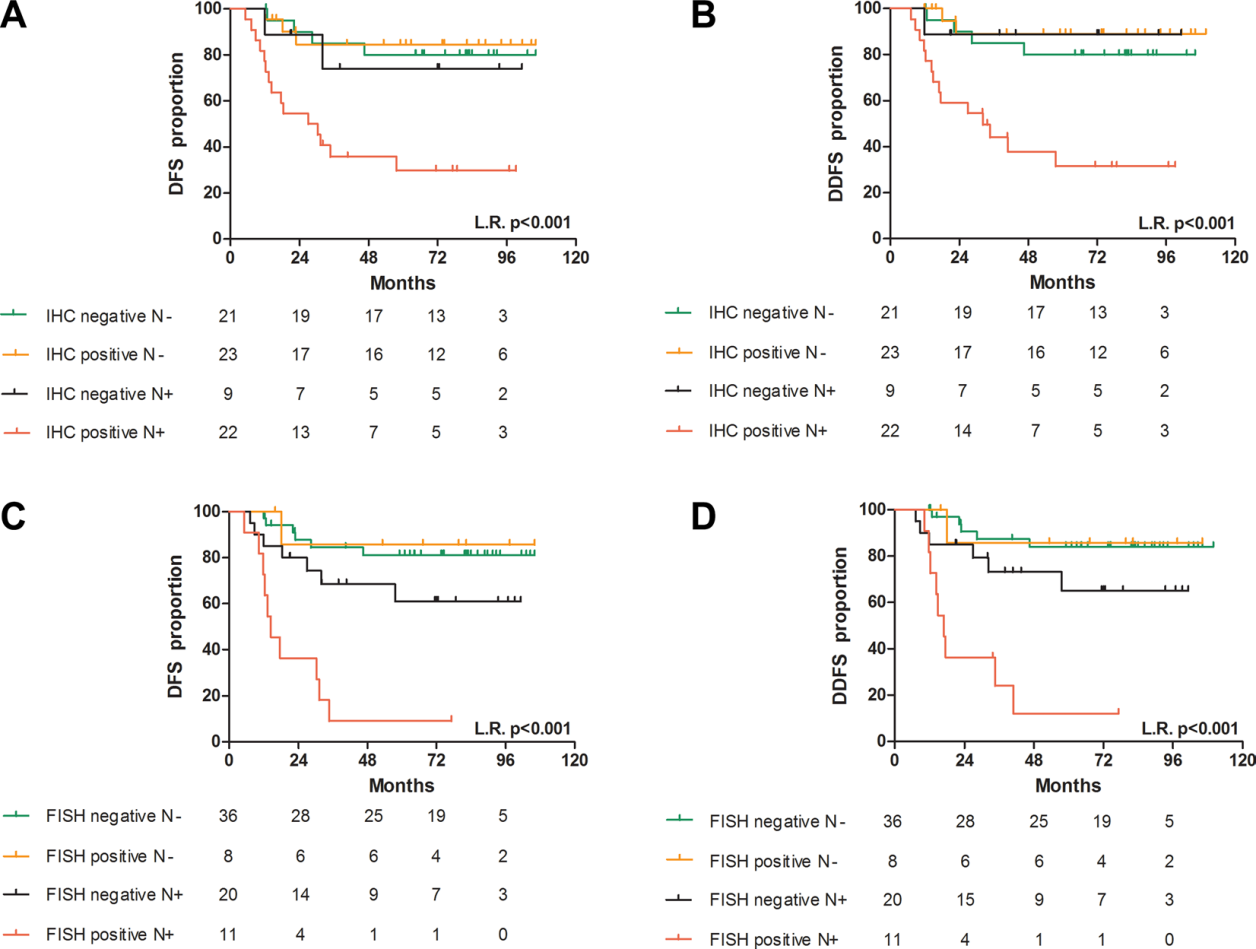
Prognostic value of CDCP1 in TNBC primary tumors Association between CDCP1 IHC status in N+ and N− patients with DFS (**A**) and DDFS (**B**) and between *CDCP1* FISH status in N+ and N− patients with DFS (**C**) and DDFS (**D**). L.R. *p* = *p*-value of the log-rank test.

Based on permutation accuracy variable importance values, as estimated using random survival forests [28], for 75 TNBC cases for which IHC and FISH data were available, we identified CDCP1 expression and *CDCP1* positivity by FISH as prognostic factors of DFS and DDFS, with age, nodal involvement, tumor size, DCIS, and Ki-67 expression (Table 3). By multivariate Cox survival analysis using the prognostic covariates above, we observed synergistic interactions between CDCP1 status by IHC and N status (Table 4, Figure 8A, 8B) and between *CDCP1* status by FISH and N status (Table 4, Figure 8C, 8D) with regard to DFS and DDFS.

**Table 3:** Unadjusted hazard ratios (HRs) by CDCP1 positivity and clinicopathological characteristics

	Disease-free survival			Distant disease-free survival		
	HR	95% CI^a^	*p*^b^	HR	95% CI^a^	*p*^b^
CDCP1 IHC-positive	2.52	1.01–6.32	0.045	2.57	0.94–7.04	0.065
CDCP1 FISH-positive	2.95	1.33–6.53	0.008	3.40	1.44–8.04	0.005
Age ≥ 50 yr	1.05	0.46–2.38	0.906	1.01	0.42–2.44	0.981
N-positive	4.59	1.90–11.11	0.001	4.65	1.80–12.06	0.002
Tumor size > 2.0 cm	2.81	1.27–6.22	0.011	2.41	1.02–5.72	0.045
DCIS ^c^	2.67	1.16–6.13	0.021	2.99	1.19–7.51	0.020
Ki-67-positive^d^	0.79	0.27–2.33	0.674	0.98	0.29–3.35	0.974

a CI, confidence interval; DCIS, ductal carcinoma *in situ.*

b Evaluated by Wald test.

c DCIS, ductal carcinoma *in situ*

d Ki-67-positive, Ki-67 > 14%.

**Table 4:** Interaction between CDCP1-IHC or -FISH status and N status in disease-free survival and distant disease-free survival

	Disease-free survival			Distant disease-free survival		
	HR (95% CI)	*p*	5-year survival	HR (95% CI)	*p*	5-year survival
IHC-N-	1.00	−	0.80 (0.55–0.92)	1.00	−	0.80 (0.55–0.92)
IHC+N-	0.85 (0.19–3.72)	0.828	0.85 (0.61–0.95)	0.40 (0.07–2.27)	0.414	0.89 (0.63–0.97)
IHC-N+	1.21 (0.21–6.84)	0.829	0.74 (0.29–0.93)	0.56 (0.06–5.06)	0.946	0.89 (0.43–0.98)
IHC+N+	5.25 (1.51–18.20)	0.009	0.30 (0.12–0.50)	3.32 (1.03–10.70)	0.044	0.31 (0.13–0.52)
FISH-N-	1.00	−	0.82 (0.64–0.91)	1.00	−	0.84 (0.66–0.93)
FISH+N-	0.76 (0.09–6.64)	0.800	0.86 (0.33–0.98)	0.69 (0.08–6.20)	0.744	0.86 (0.33–0.98)
FISH-N+	2.03 (0.64–6.51)	0.231	0.61 (0.34–0.80)	1.87 (0.53–6.59)	0.332	0.65 (0.37–0.83)
FISH+N+	8.89 (2.64–29.92)	< 0.001	0.09 (0.01–0.33)	7.18 (2.09–24.65)	0.002	0.12 (0.01–0.40)

Adjusted hazard ratios (HRs) from a Cox multivariable model (with age, tumor size, Ki-67 levels, and DCIS as additional covariates); significance (*p*) of the HRs and estimated 5-year survival are listed.

The probability of developing distant metastases at 5 years of follow-up in the N-positive subgroup was 69% for CDCP1 IHC-positive patients and 11% in CDCP1 IHC-negative patients (*p* = 0.0246), whereas in N-negative patients, the likelihood was 11% and 20%, respectively (*p* = 0.450) (Figure 8B). Similarly, the probability of developing distant metastases at 5 years in the N-positive subgroup was 88% for *CDCP1* FISH-positive cases versus 35% for negative patients (*p* = 0.004) but 14% and 16% in N-negative/*CDCP1* FISH-negative and -positive patients, respectively (*p* = 0.935) (Figure 8D). The hazard ratios (HRs) of the synergistic interaction of CDCP1 IHC and FISH positivity with N positivity in predicting disease relapse did not differ significantly (*p* = 0.9478 for DFS and *p* = 0.7115 for DDFS).

## DISCUSSION

We have identified CDCP1 as a valuable marker of TNBC aggressiveness, implicating it as a novel potential therapeutic target, favored by its transmembrane localization, which renders it easily accessible by drugs. Our study demonstrates that the transmembrane receptor CDCP1 is overexpressed in ~60% of human primary TNBCs, 38% of which exhibit a gain in *CDCP1* copy number by FISH analysis, and that CDCP1 overexpression is linked to shorter DFS and DDFS in TNBC.

The rise in CDCP1 mRNA and protein levels in TNBC cell lines on treatment with postsurgery WHFs, which mimic a protumor microenvironment, implicates CDCP1 in driving the aggressiveness of this tumor subtype. Despite mRNA and protein levels increasing in response to WHF, the extent to which they did so differed, possibly due to post-transcriptional mechanisms that regulate CDCP1 expression [29, 30] or the localization of CDCP1 to poorly soluble cell membrane compartments [31]. These mechanisms might also differentially affect CDCP1 levels between cell lines. Unlike the 135-kD CDCP1 species, no increase in the 70-kD membrane-bound form was observed in most cell lines, suggesting that the WHF primarily regulates the full-length form, likely through its neosynthesis. In BT-549 and HCC1937 cells, in which the 70-kD form was downregulated only slightly after WHF treatment, the increase in the full-length form was nonetheless greater than the reduction in the 70-kD form, suggesting that the higher levels of full-length CDCP1 do not account for the reduction in its cleavage.

As reported for the MDA-MB-231, T47D, and MDA-MB-468 breast cancer cell lines [32, 21], CDCP1 expression and their migration and invasion *in vitro* were significantly associated. Moreover, CDCP1 promoted the formation of vascular-like channels, a property of TNBC that correlates with lower DFS [27], further implicating it in the acquisition of a more malignant phenotype. Notably, CDCP1 knockdown was associated with a decrease in the phosphorylation of canonical molecules that read out the activity of CDCP1 (Src and PKCδ) [26] in MDA-MB-231 but not BT-549 cells, suggesting that CDCP1-driven aggressiveness in TNBC cells could also be independent of canonical downstream molecules.

With regard to cell proliferation, we observed a negligible effect of CDCP1 on adhesion-dependent cell growth but confirmed the correlation between CDCP1 and anchorage-independent growth in TNBC [21]. This effect might depend on CDCP1-dependent resistance to anoikis [12], which mediates growth *in vitro* in 3D cultures and *in vivo*, rather than on its involvement in proliferation. Accordingly, whereas a study of 25 cases of breast cancer specimens, including hormone receptor-positive and -negative cases, reported that most tumors with high *CDCP1* mRNA levels were positive for the proliferation marker Ki-67 [33], we found no association between CDCP1 expression and proliferation rates in our tumor series.

Consistent with our *in vitro* findings, TNBC patients with CDCP1-positive specimens had a higher risk of distant relapse. The prognostic value of CDCP1 has been reported in human cancers, wherein CDCP1 overexpression is associated with a poor prognosis in certain epithelial tumors [13–17, 20], but our study is the first description of its clinical significance in TNBC patients.

Our analyses in human TNBC specimens also revealed an interaction between CDCP1 and nodal status, whereby CDCP1 expression identified cases that were at high risk of developing distant metastases, suggesting that only CDCP1-positive TNBCs among tumors that have already disseminated at the time of surgery tend to relapse. The similar frequencies of CDCP1-positive cases in the N-positive and -negative subgroups in our series raise the possibility that CDCP1 expression in disseminated TNBC accelerates the metastatic process. In N-positive TNBCs, for example, CDCP1 might promote transendothelial migration of TNBC cells or confer properties to tumor cells that have disseminated that improve their survival in circulating blood. This hypothesis is supported by the ability of CDCP1 to effect resistance to anoikis [12] and by the association between CDCP1 expression and the development of distant metastases in small tumors, such as those in our series.

The gain in *CDCP1* copy number in 38% of CDCP1-positive TNBC cases by FISH and the evidence that tumors with a high number of cells that express CDCP1 also bear many cells with alterations in *CDCP1* support that a genetic gain in *CDCP1* in this breast cancer subtype is involved in CDCP1 expression. Nevertheless, other mechanisms might govern CDCP1 expression in TNBC primary tumors without such gains, such as those in tumor hypoxia in renal cancer cells [34] and hepatocellular carcinoma [35], the EGF/EGFR pathways in ovarian models [36], BMP4 in pancreatic cells [37], and unidentified molecules in the tumor microenvironment of primary TNBCs, as suggested by WHF treatment.

The absence of an association of CDCP1 expression and copy number gains with clinicopathological features or the expression of a basal-like BC marker, such as cytokeratin 5/6 [38], suggests that CDCP1 is already present in early-stage TNBCs, independent of the molecular phenotype of tumor cells. Because mesenchymal-like and basal-like TNBCs have similar outcomes [4], their aggressiveness could account for their commonality, including their expression of CDCP1.

While our findings await validation in larger TNBC series, our data demonstrate the value of CDCP1 in predicting the aggressiveness of TNBC tumors and identify a therapeutic target for this disease.

## MATERIALS AND METHODS

### Cell lines, cultures, and treatments

The human breast cancer cell lines MDA-MB-231, BT-549, MDA-MB-157, HCC1937, MDA-MB-468 (American Type Culture Collection), SUM149, and SUM159 (Asterand Bioscience, Detroit, MI) were authenticated using a panel of microsatellite markers. Cell lines were maintained at 37°C in a humidified atmosphere of 5% CO_2_ in air as follows: MDA-MB-231 and BT-549 in RPMI 1640 (Sigma-Aldrich); MDA-MB-468 in Dulbecco's modified Eagle's medium (DMEM) (Lonza); MDA-MB-157 in Leibovitz (Lonza); SUM149 and SUM159 in DMEM F12 (Lonza) that was supplemented with insulin (5 μg/ml); and HCC1937 in RPMI 1640 medium that was supplemented with 1 mM sodium pyruvate, 1% (v/v) nonessential amino acids, and 10 mM Hepes. Each medium was also supplemented with 10% fetal bovine serum (FBS) and 2 mM glutamine (both from Sigma-Aldrich). For the stimulation with WHF, cells were starved in serum-free medium for 24 h and then treated for 48 h with a pool of 5 WHFs at a final concentration of 5% as described [24].

### Antibodies

In the biochemical analyses, we used rabbit polyclonal antibody against CDCP1 (Merck Millipore); rabbit polyclonal phospho-CDCP1 (Tyr734) (Cell Signaling); mouse monoclonal anti-Src, clone GD11 (Merck Millipore); rabbit polyclonal phospho-Src family (Tyr416) (Cell Signaling); rabbit polyclonal anti-PKCδ (Cell Signaling); rabbit polyclonal phospho-PKCδ (Tyr311) (Cell Signaling); mouse monoclonal anti-vinculin, clone hVIN-1 (Sigma); anti-rabbit or -mouse IgG (GE Healthcare) as the secondary antibody; and peroxidase-linked mouse monoclonal anti-actin (Sigma-Aldrich). In the IHC analyses, we used CDCP1 polyclonal antibody PA5-17245 (Thermo Fisher Scientific).

### Western blot

To prepare crude cell lysates, cells were processed as described [39]. Protein concentrations were determined by Coomassie Plus protein assay (Thermo Fisher Scientific). The samples were separated on NuPage SDS-Bis-Tris gels (Life Technologies) and transferred onto PVDF membranes (EMD Millipore). Signals were detected using ECL reagent (GE Healthcare). Densitometric analysis was performed using Quantity One 1-D (Bio-Rad), with CDCP1 expression normalized to that of actin.

### Growth, migration, and invasion *in vitro* assays

Relative 2D cell growth was measured by sulforhodamine B (SRB) assay [40]. Optical density was determined on an ELISA microplate reader (Bio-Rad Laboratories). Anchorage-independent tumor growth (3D) was assessed in cells that were suspended in complete medium that contained 0.2 % agar and seeded in 24-well plates at 2500 cells/well on a 0.5–ml base of complete medium that contained 0.4% agar; colony growth was examined by counting all colonies in each well 15 days after seeding at 40× magnification.

To evaluate migration, cells were seeded in the top of a Boyden chamber (Sigma-Aldrich) in serum-free medium, and medium with 10% FBS was placed in the well below; a Matrigel layer (Basement Membrane Matrix; BD Bioscience) was added for the invasion assay. Migration and invasion by MDA-MB-231 cells was determined as the area that they occupied at 12 h, whereas for BT-549 cells, these analyses were performed at 6 h. At the end of the incubation, cells in the upper chamber were removed with cotton swabs, and those that traversed the Matrigel were fixed in 100% ethanol, stained with SRB, and imaged on an ECLIPSE TE2000-S inverted microscope (Nikon Instruments). The results were expressed as the area that was occupied by cells on the bottom of the Transwell, as evaluated by digital image analysis using the appropriate software (Image Pro-Plus 7.0 application, Media Cybernetics) in 3 independent experiments (± SEM). Differences were considered to be significant at *p* < 0.05.

### *In vitro* tube formation assay

Cells (2 × 10^4^) were seeded in 96-well plates that were precoated with Matrigel (Corning) (35 μl/well, diluted 1:1 in medium without FBS) and incubated for 2–4 h at 37°C. Tube formation was detected using an EVOS^®^ XL Core Cell Imaging Systems inverted light microscope (AMG) (10× magnification) as described [27].

### Knockdown of CDCP1 by siRNA transfection

Knockdown of CDCP1 with siRNA (ON-TARGET plus SMART pool, GE Healthcare) was performed with a pool of 4 oligonucleotides that could bind and degrade *CDCP1* mRNA and were tested for silencing efficiency and the presence of off-targets.

Negative control: 5′ UGGUUUACAUGUCGACU AAdTdT3′

*CDCP1*-1:5′AGGAGGAGCGGGUUGAAUAdTdT3′

*CDCP1*-2:5′CCACGAGAAAGCAACAUUAdTdT3′

*CDCP1*-3:5′CCAGAAAUGUCUCCGGCUUdTdT3′

*CDCP1*-4:5′GAGCAUCGGUUUAGAGCUGdTdT3′

Cells were transfected with CDCP1 siRNAs (100 nM) using RNAiMAX (Life Technologies) or with the appropriate control siRNAs (On-TARGETplus Non-Targeting Pool, GE Healthcare), harvested at 48 h posttransfection, and examined for CDCP1 expression by western blot. Densitometric analysis was performed using Quantity One 1-D (Bio-Rad), with CDCP1 expression normalized to that of actin. The knockdown efficiency, expressed as the percentage reduction in CDCP1 expression that was induced by CDCP1 siRNA compared with the control siRNA, was ≥ 80%, considering both CDCP1 forms. Silencing of CDCP1 in MDA-MB-231 and BT-549 cells was stable for more than 10 days (data not shown).

### Gene expression analysis

Total RNA was isolated from samples using Qiazol (Qiagen). RNA integrity and purity were assessed on a Bioanalyzer (Agilent) after total RNA *in vitro* amplification; the RNA was then labeled with biotin and analyzed on Illumina HumanHT-12 V3.0 expression beadchips (Illumina). This array contains over 47,000 transcripts that are derived from the UniGene database. Array chips were washed per the manufacturer's protocol, stained with 1 μg/ml Cy3-streptavidin (GE Healthcare), and scanned on an Illumina BeadArray Reader. Intensity values were quality-checked, and the dataset was normalized using the quantile algorithm and BeadStudio, version 3. For each gene, the detection value was set to *p* < 0.05, and 50% of missing values was the cutoff to filter reliable data. All microarray data are MIAME-compliant, and the raw data were deposited into the NCBI Gene Expression Omnibus (GEO) database (http://www.ncbi.nlm.nih.gov/projects/geo/) under accession number GSE59614.

### Bioinformatics analysis of microarray data

WHF-treated and untreated MDA-MB-231, BT-549, MDA-MB-468, SUM149, and MDA-MB-157 cells were split into training and validation sets. The experimental settings were designed, considering a biological replicate of 5 paired (treated and untreated) TNBC cell lines. The data were validated in an independent preparation of the 5 cell lines following the same experimental design. Differentially expressed genes were imputed by class comparison, based on a false discovery rate (FDR) < 0.1 and following a paired experimental design in the training set.

Differentially expressed genes were then tested in the validation set. In this dataset, 21,192 of 47,000 transcripts were detected, and 471 Illumina probe sets that corresponded to 427 unique genes were differentially expressed: 175 probes were upregulated in WHF-treated cells versus 296 in untreated cells, indicating that WHF modulated gene expression under our working conditions. Gene-set enrichment analysis was performed using GSEA v2.0.13 [41] on GO biological processes. GSEA was run using a pre-ranked gene list according to the t statistic obtained from differential expression analysis. Because we aimed to identify plasma membrane receptors that were upregulated in TNBC models and associated with TNBC aggressiveness as potential therapeutic targets, our analyses were restricted to 796 membrane surface molecules that are expressed in all human tissues. This list was generated per Castellano et al. [42] and updated by a manually curated PubMed search and integration with HPMR [43], with the database provided by Almén [44]; 258 of the 796 unique genes were detected in our breast cancer models.

At a significance threshold of FDR < 0.1 and fold-change > 2, only one gene, *CDCP1*, was differentially upregulated. *CDCP1* mRNA levels were analyzed using the best-performing probe, ILMN_1708167, as indicated by Illumina Inc.; the 4.17-fold change in mRNA that we have reported is derived from this analysis, which considered all TNBC cell lines, and represents the geometric mean log fold-change in *CDCP1* mRNA (WHF-treated *vs* untreated cells). A TaqMan assay that specifically amplified *CDCP1* transcripts (Hs01080405_m1, Hs01080410_m1; Thermo Fisher Scientific) was performed to confirm the statistically significant increase in *CDCP1* mRNA levels that were observed in the gene expression analysis.

### Patients and WHFs

Samples from 100 TNBC patients who were diagnosed between August 2002 and February 2007 in our institute (Fondazione IRCCS Istituto Nazionale dei Tumori) were selected, based on IHC criteria (<1% of cells positive for estrogen receptor, progesterone receptor, and HER2 expression, classified as 0 or 1+) and availability of follow-up. Twelve WHFs from breast cancer patients who were diagnosed in 2010–2011 and did not undergo neoadjuvant therapy, without a high glycemic index (> 110 mg/dl), were collected from the first clearing of surgical closed-type breast drains (no abdomen or armpit) under suction during the 24 h postsurgery. WHFs were centrifuged immediately at 3000 g, aliquoted, and stored at −80°C. The protein concentration in the WHFs, as determined by Biureto method, ranged from 3.7 to 5.1 g/dl. WHFs were pooled, 5 at a time in each experiment, and used at a final concentration of 5% in medium, wherein each WHF had a final concentration 1%, as in our previous work [24]. WHFs in medium were passed through a 0.22–μm syringe PVDF filter (Merck-Millipore) before cell stimulation.

Supplementary Table S1 lists the pathobiological characteristics of the patients from which the WHFs were derived. All patients gave written consent for use of their biological materials for future investigations and research purposes, and the study did not require further institutional approval from the ethics committee. All data were analyzed anonymously, and all experiments complied with the Declaration of Helsinki. The median follow-up of the cohort of 100 patients was 5.4 years.

### Immunohistochemistry

Expression of CDCP1 was analyzed by IHC on consecutive 2-μm formalin-fixed, paraffin-embedded (FFPE) tumor sections, using rabbit polyclonal anti-CDCP1 (1:50) (PA5-17245, Thermo Fisher Scientific), which is directed against the C-terminus of CDCP1, after antigen retrieval, which as performed by heating the sections for 5 min at 96°C in 10 mM citrate buffer, pH 6.0. Immunoreactions were visualized using streptavidin-biotin-peroxidase (Dako, Agilent Technology, Santa Clara, CA), 3,3′-diaminobenzidine (DAB; brown signal) (Dako) as the chromogen, and the sections were counterstained with hematoxylin. Images were acquired on an ECLIPSE TE2000-S inverted microscope (Nikon Instruments, Melville, NY) at 20× and 40× magnification.

The reactivity of anti-CDCP1 in the TNBC specimens was considered to be positive when ≥ 10% of tumor cells showed membrane staining. This cutoff was chosen, based on distribution analysis of the percentage of CDCP1-positive cells by IHC in each tumor section. No tumors had < 10% CDCP1-positive cells in our series, and cases with different percentages of CDCP1-positive cells were likewise distributed in a 10–100% interval. By explorative Kaplan-Meyer analysis of DFS in our cases—stratified as negative, ≥ 10% and < 50%, or ≥ 50% for CDCP1 expression, both CDCP1-positive groups had a worse and superimposable DFS compared with CDCP1-negative cases (data not shown).

### Fluorescence *in situ* hybridization (FISH)

For FISH studies, 3 bacterial artificial chromosome (BAC) clones (BAC 2311L2, spanning only *CDCP1*; and BAC 265303 and BAC 3050I8, covering the CDCP1 region and *CLEC3B* and *TMEM158* on 3p21.31), chosen from Genome Browser of the University of California of Santa Cruz (http://genome.ucsc.edu/), were used. Each clone was tested by FISH in metaphase cells to confirm its location on 3p21.31 and to grade their signal strength and specificity. A commercial probe for centromere 3, CEP3 (Abbott Molecular), was mixed with the BAC pool to better assess the presence of genetic alterations, such as amplification, deletion, and polysomy. To improve the FISH signal intensity, the mixture of BACs was used after verifying that the percentage of positive cells remained identical to that obtained using only BAC 2311L2 in a pilot cohort of tumors (20 TNBCs). The pooled BACs were labeled with Spectrum Green (Abbott Molecular, Illinois) using a nick-translation kit (Abbott Molecular).

A representative example of the FISH results with BAC 2311L2 alone or the mixture of BACs is provided in Supplementary Figure S2. In light of the heterogeneity of TNBCs, the mean signal per tumor cell for CDCP1 and CEP3 was recorded for each case, as was the percentage of tumor cells with an alteration in gene copy number. For the amplified CDCP1 and polysomic CDCP1 categories, we used cutoffs of 10% and 40% positive cells, respectively—the same criteria that have been used for the analysis of HER2 [45], EGFR [46], and MET [47], the prognostic values of which are associated with a genetic acquisition. All FISH analyses were performed on FFPE tissues in areas that were selected by the pathologist as CDCP1-positive by IHC or, for IHC-negative cases, representative of the tumor.

### Statistical analysis

Relationships between categorical variables were analyzed by Fisher's exact test. Two-tailed Student's *t*-test was used to compare mean values of 2 independent groups; for 2 dependent groups, the equality of means was examined by two-tailed paired *t*-test. An approach that was based on minimal depth variable importance, as estimated by random survival forests [28], was used to select prognostic factors of DFS and DDFS. Kaplan-Meier survival curves and exact log-rank tests were used to analyze differences in DFS and DDFS between 2 or more tumor groups. Adjusted HRs of prognostic factors and their 95% confidence intervals (CIs) were estimated by fitting multivariable Cox survival models. The null hypothesis that a hazard ratio (HR) estimated in a Cox model is equal to 1 was evaluated using the Wald test. Differences were considered to be significant at *p* ≤ 0.05. All analyses were performed using Stata 13.0 (Stata) and R 3.13.2.

## SUPPLEMENTARY MATERIALS


