# Supplementary material for: CDCP1 is a novel marker of the most aggressive human triple-negative breast cancers
Source: Oncotarget. 2016 Sep 10;7(43):69649–65. doi: 10.18632/oncotarget.11935 (PMC5342505; doi:10.18632/oncotarget.11935)
Supplement: Supplementary file 1 [file oncotarget-07-69649-s001.pdf]

CDCP1 is a novel marker of the most aggressive human triple-negative breast cancers

Supplementary Materials

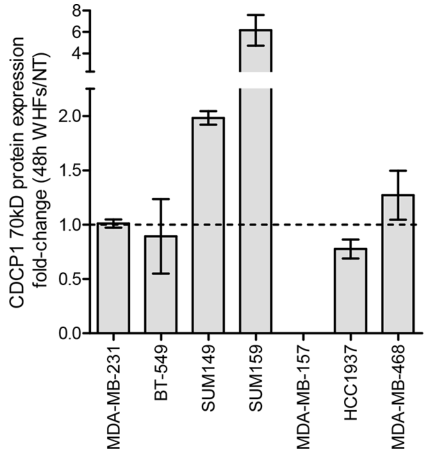

Supplementary Figure S1: WHF regulation of cleaved membrane-bound CDCP1 expression. Shown are the fold-increases (± SEM) in the cleaved membrane-bound CDCP1 protein (70-kD) upon WHF stimulation of each cell line in at least 3 independent Western blot experiments with respect to untreated cells. Densitometric analysis was performed as described in Materials and Methods; CDCP1 70-kD expression levels were normalized on actin levels.

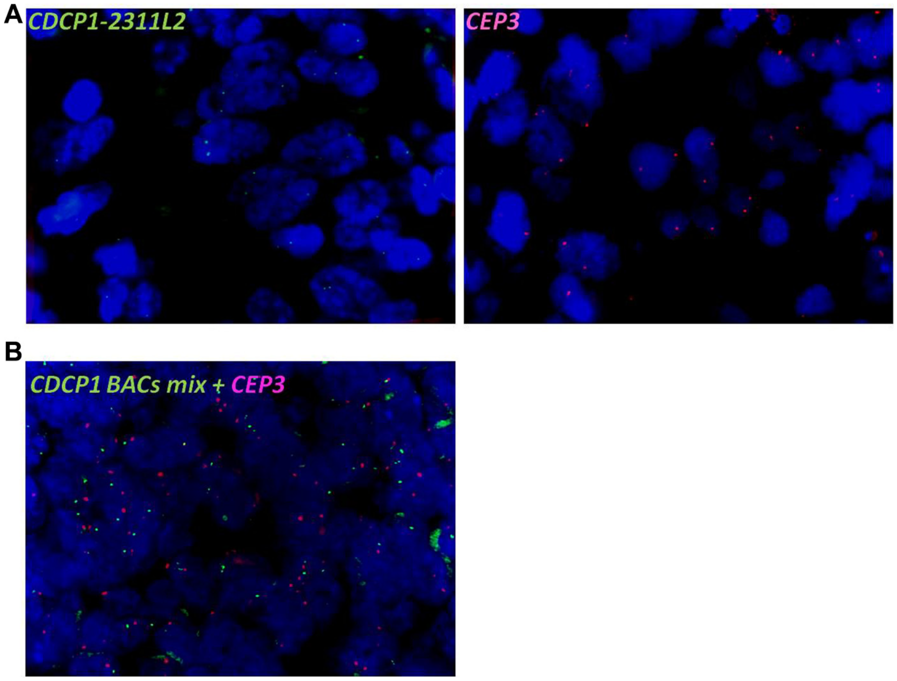

Supplementary Figure S2: Representative images of FISH using CDCP1-2311L2 probes, spanning only the CDCP1 gene, and CEP3 probe (**A**) or a mix of three BACs (BAC 2311L2, BAC 265303 and BAC 3050I8) and CEP3 probe (**B**) on the same FFPE human primary TNBC specimen. This case is polysomyc for CDCP1 gene.

Supplementary Table S1: Tumor clinicopathological characteristics of patients from whom WHFs were collected

Patient ID	Age (yr)	Histotype[a]	Size (cm)	Grade	N status[b]	ER[c]	PgR[d]	HER2 status
#1	70	IDC+ILC	1.1	2	neg	pos	pos	0
#2	68	IDC	3.5	3	neg	neg	neg	0
#3	42	Mucinous IDC	3.5	2	pos	pos	neg	3+
#4	68	IDC	1.5	3	pos	pos	pos	2+
#5	71	IDC+ILC	1.2	2	neg	pos	pos	1+
#6	45	IDC	1.5	3	pos	pos	pos	1+
#7	61	DCIS	2	2	neg	pos	pos	1+
#8	77	Tubular IDC	1.5	1	neg	pos	neg	1+
#9	43	IDC	0.8	3	nd	neg	neg	1+
#10	33	Mucinous IDC	2.5	3	pos	pos	neg	3+
#11	56	IDC	2.5	3	pos	neg	neg	1+
#12	90	IDC	2.2	3	neg	pos	neg	3+

[a]IDC, invasive ductal carcinoma; ILC, invasive lobular carcinoma; DCIS, ductal carcinoma *in situ*.

[b]nd, not determined.

[c]ER-pos, > 10% cell positivity.

[d]PgR-pos > 10% cell positivity.
